# Gamma-aminobutyric acid and glutamate/glutamine levels in the dentate nucleus and periaqueductal gray with episodic and chronic migraine: a proton magnetic resonance spectroscopy study

**DOI:** 10.1186/s10194-022-01452-6

**Published:** 2022-07-15

**Authors:** Wei Wang, Xueyan Zhang, Xiaoyan Bai, Yingkui Zhang, Ziyu Yuan, Hefei Tang, Zhiye Li, Zhangxuan Hu, Yaqing Zhang, Xueying Yu, Binbin Sui, Yonggang Wang

**Affiliations:** 1 Headache Center, Department of Neurology, Beijing Tiantan Hospital, Capital Medical University, No.119 South Fourth Ring West Road, Fengtai District, Beijing, 100070 China; 2 Tiantan Neuroimaging Center of Excellence, China National Clinical Research Center for Neurological Diseases, No.119 South Fourth Ring West Road, Fengtai District, Beijing, 100070 China; 3 Department of Radiology, Beijing Tiantan Hospital, Capital Medical University, No.119 South Fourth Ring West Road, Fengtai District, Beijing, 100070 China; 4 Department of Neurology, The First Affiliated Hospital of Zhengzhou University, No.1, Jianshe East Road, Zhengzhou, Henan Province 450000 China; 5 GE Healthcare, No.1 Tongji Nan Road, Beijing Economic Technological Development Area, Beijing, 100070 China

**Keywords:** Magnetic resonance spectroscopy, Migraine chronification, Gamma-aminobutyric acid, Glutamate/glutamine, Dentate nucleus, Periaqueductal gray

## Abstract

**Background:**

The pathogenesis of migraine chronification remains unclear. Functional and structural magnetic resonance imaging studies have shown impaired functional and structural alterations in the brains of patients with chronic migraine. The cerebellum and periaqueductal gray (PAG) play pivotal roles in the neural circuits of pain conduction and analgesia in migraine. However, few neurotransmitter metabolism studies of these migraine-associated regions have been performed. To explore the pathogenesis of migraine chronification, we measured gamma-aminobutyric acid (GABA) and glutamate/glutamine (Glx) levels in the dentate nucleus (DN) and PAG of patients with episodic and chronic migraine and healthy subjects.

**Methods:**

Using the MEGA-PRESS sequence and a 3-Tesla magnetic resonance scanner (Signa Premier; GE Healthcare, Chicago, IL, USA), we obtained DN and PAG metabolite concentrations from patients with episodic migraine (*n* = 25), those with chronic migraine (*n* = 24), and age-matched and sex-matched healthy subjects (*n* = 16). Patients with chronic migraine were further divided into those with (*n* = 12) and without (*n* = 12) medication overuse headache. All scans were performed at the Beijing Tiantan Hospital, Capital Medical University.

**Results:**

We found that patients with chronic migraine had significantly lower levels of GABA/water (p = 0.011) and GABA/creatine (Cr) (p = 0.026) in the DN and higher levels of Glx/water (p = 0.049) in the PAG than healthy controls. In all patients with migraine, higher GABA levels in the PAG were significantly associated with poorer sleep quality (GABA/water: *r* = 0.515, *p* = 0.017, *n* = 21; GABA/Cr: *r* = 0.522, *p* = 0.015, *n* = 21). Additionally, a lower Glx/Cr ratio in the DN may be associated with more severe migraine disability (*r* = -0.425, *p* = 0.055, *n* = 20), and lower GABA/water (*r* = -0.424, *p* = 0.062, *n* = 20) and Glx/Water (*r* = -0.452, *p* = 0.045, *n* = 20) may be associated with poorer sleep quality.

**Conclusions:**

Neurochemical levels in the DN and PAG may provide evidence of the pathological mechanisms of migraine chronification. Correlations between migraine characteristics and neurochemical levels revealed the pathological mechanisms of the relevant characteristics.

**Supplementary Information:**

The online version contains supplementary material available at 10.1186/s10194-022-01452-6.

## Background

Migraine is a common disabling brain disorder that affects approximately 14% of the general population worldwide (males 8.6%, females 17.0%) [1]. This disease is typically characterized by moderate or severe headaches, and patients often experience additional symptoms such as nausea, phonophobia, and photophobia that last from 4 to 72 h. According to the International Classification of Headache Diseases, 3rd edition (ICHD-3) diagnostic criteria, migraine is divided into episodic migraine (EM) and chronic migraine (CM) [2]. Most patients have EM; however, up to 5% of patients may develop CM (headache occurring 15 or more days per month for more than 3 months that have the features of migraine headache at least 8 days per month) [2]. At least 50% of patients with CM regularly overuse one or more drugs for acute migraine treatment, thus resulting in the diagnosis of CM with medication overuse headache (MOH) [3]. CM often affects people during their most productive years of life, exerts substantial individual and societal costs, and is associated with numerous comorbid disorders [4]. The pathogenesis of CM and the mechanisms leading to its transformation remain unclear. Current theories include atypical pain processing, genetic and epigenetic factors, central sensitization, cortical hyperexcitability, and neurogenic inflammation [5]. Genetic studies have revealed the potential involvement of glutamatergic and GABAergic receptors in the pathogenesis of migraine [6–8]. Additionally, some studies have shown that hyperexcitability in the cortex [9–13], suggests that an unbalanced inhibition-excitation system in the brain contributes to the pathophysiology of migraines. However, the specific roles of glutamate and glutamine (Glx) and gamma-amino-butyric acid (GABA), which are the major excitatory and inhibitory neurotransmitters in the brain, in migraine are not fully understood.

During the past two decades, numerous structural and functional magnetic resonance imaging (MRI) studies have shown that the pathological mechanism of migraine is closely related to abnormalities in the central nervous system [14]. Magnetic resonance spectroscopy (MRS) is a noninvasive method that allows the investigation of brain metabolism. Several studies have used MRS to investigate metabolic changes in the brain regions and nuclei of patients with migraine [15]. The periaqueductal gray (PAG) is one of the most significant elements of the endogenous descending modulatory system, and numerous MRI studies have shown that the PAG has structural and functional abnormalities in patients with migraine [14, 16, 17]. The cerebellum has been implicated in various forms of motor control and coordination; however, more recently, it has also been suggested to have a role in nonmotor functions, including cognition and pain processing [18]. Currently, Glx and GABA in the cerebellum and PAG of patients with migraine have not been studied. Therefore, we used proton MRS (^1^H-MRS), specifically the MEGA-PRESS technique, to explore the GABA and Glx levels in the PAG and dentate nucleus (DN), which is the largest nucleus in the cerebellum, of patients with EM and CM. Our primary aim was to determine whether there is an association between changes in GABA and Glx levels and EM and CM to further elucidate the role of neurotransmitters in migraine chronification. The secondary aim was to explore the differences in GABA and Glx levels of patients having CM with and without MOH to further elucidate the effects of medication overuse on neurotransmitters.

## Methods

### Study design

This study used a cross-sectional, case–control design and MRS to measure GABA and Glx levels of patients with EM and CM and healthy control subjects. Ethical approval was granted by Beijing Tiantan Hospital, Capital Medical University (no. KY2022-044). Written consent was obtained from participants in accordance with the principles of the Declaration of Helsinki.

### Participants

We prospectively recruited 22 healthy subjects and 62 patients diagnosed with EM (*n* = 29) and CM (*n* = 33) from October 2020 to December 2021 at the Headache Center, Department of Neurology, Beijing Tiantan Hospital, Capital Medical University. EM and CM (with and without MOH) were diagnosed according to the ICHD-3 [19]. All patients had migraine without aura. MOH is defined as a complication of CM which regular overuse of drugs for the acute treatment of headache. To establish the diagnosis, patients have to use symptomatic headache medication on more than 10 or more than 15 days per month, depending on the drug class, for more than 3 months [20–22]. Demographics, headache characteristics, body mass index, migraine disability assessment scale (MIDAS) scores, Patient Health Questionnaire-9 (PHQ-9) scores, Generalized Anxiety Disorder-7 (GAD-7) scores, Pittsburgh Sleep Quality Index (PSQI) scores, and Montreal Cognitive Assessment (MoCA) scores were recorded by our research questionnaire before the MRS study. The MIDAS is used to measure headache-related disability, PHQ-9 is designed to measure symptoms of depression in primary care settings, GAD-7 is used to assess anxiety, PSQI is an effective instrument used to measure the quality and patterns of sleep, and the MoCA is a means of accurately detecting levels of cognitive impairment. The inclusion criteria for participants were as follows: age 14 to 60 years; feasibility of MRS performance (for example, no claustrophobic syndrome and no metal in the body); and complete data were available. The exclusion criteria were as follows: headache directly related to secondary factors existing at enrollment according to the ICHD-3 diagnostic criteria; migraine with other types of primary headache; other diseases such as musculoskeletal disorders and rheumatism that may lead to overuse of analgesics; poor MRS data quality; and imprecise diagnosis.

### Magnetic resonance imaging and spectroscopy

**Fig. 1:**
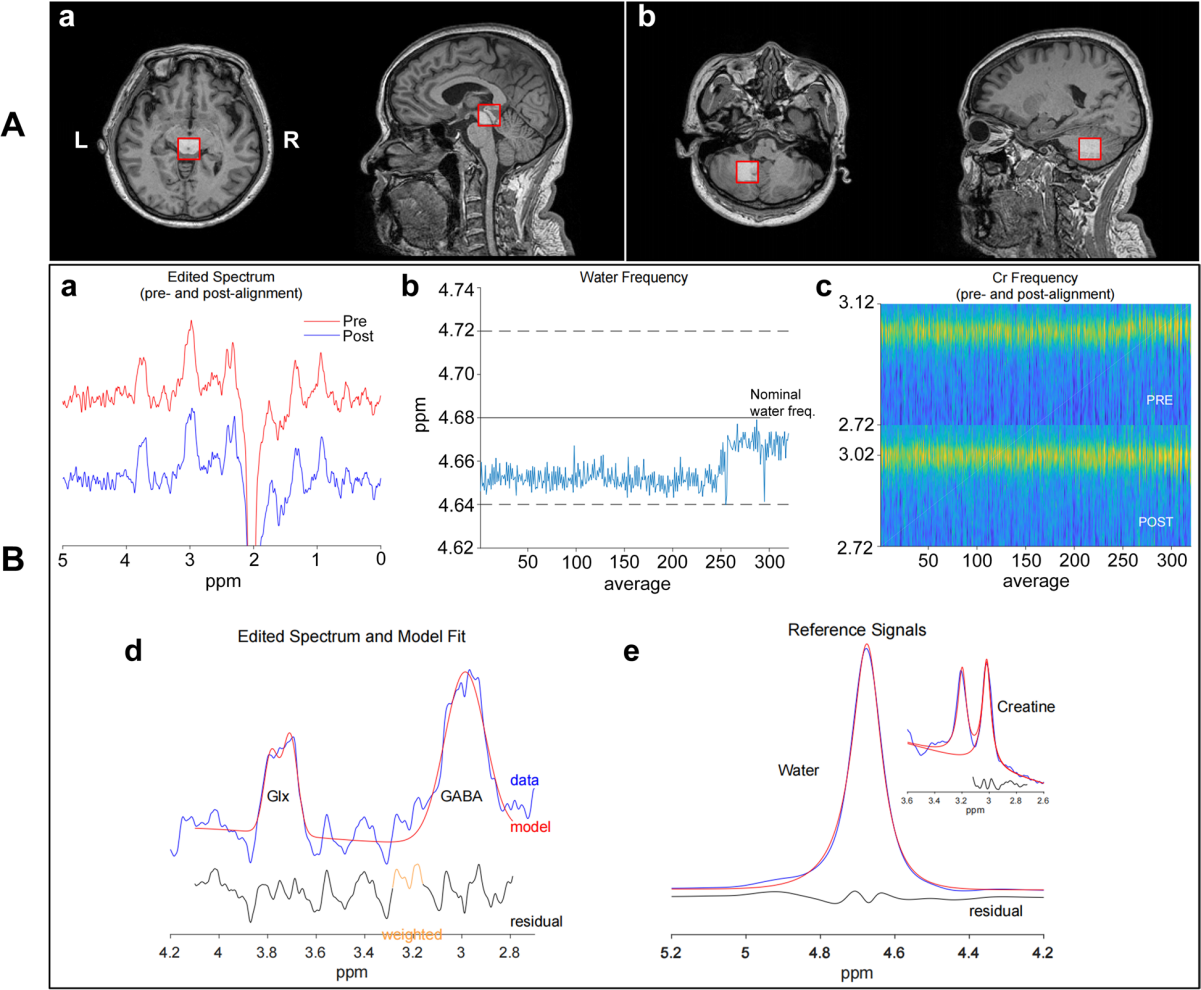
Magnetic resonance spectroscopy voxel placement and data processing. **A** This schematic diagram demonstrates the voxel placement in the periaqueductal gray (PAG) (**a**) and dentate nucleus (DN) (**b**). The red lines represent the region of interest. **B** The processed gamma-aminobutyric acid (GABA)-edited difference spectrum **(a**) before frequency and phase correction (red) and (**b**) after (blue). The residual water signal is plotted against time, providing qualitative information about the stability of the experiment The creatine (Cr) signal over the duration of the experiment is shown (**c**). The y-axis represents the frequency (in ppm) of the Cr signal. The modeling of the GABA signal is shown (**d**). The GABA-edited spectrum is shown in blue. The red represents the model of best fit. The residual between these two is shown in black. Modeling of the signal against which GABA is quantified is shown (**e**). Data are shown in blue. Models are shown in red. Residuals are shown in black. L, left; R, right

MR imaging was performed on a 3 T MR scanner (Signa Premier, GE Healthcare) using a 48-channel head coil. Patients were instructed to keep their head and neck stable, stay awake, close the eyes, and relax during the magnetic resonance (MR) scans. Scanning was performed using a 3-T MR scanner (Signa Premier, GE Healthcare) and a 48-channel head coil. T1-weighted volumetric images were acquired using the MP-RAGE sequence with 1-mm isotropic resolution (sagittal acquisition: field of view, 256 mm; acquisition matrix, 256; slice number, 192; flip angle, 8°; preparation time, 880 ms; recovery time, 400 ms; acceleration factor, 2*;* acquisition time, 4:00). Two 20 × 20 × 20 mm^3^ voxels were placed in PAG (Fig. 1Aa) and DN (Fig. 1Ab) respectively. The ^1^H spectrum optimized for detecting GABA was acquired individually for these voxels using the MEGA-PRESS sequence with the following parameters: echo time/repetition time, 68/2000 ms; number of points, 2048; spectral width, 2000 Hz; and number of averages, 160 (scan time, 11 min 28 s). The MEGA-PRESS data were analyzed using the GABA analysis toolkit (GANNET3.1; http://gabamrs.org) [23], which uses a Gaussian baseline model to fit the edited GABA signal and a Lorentz-Gaussian line shape to fit the unsuppressed water signal. The processing steps were as follows: combination of phased array coil data; time-domain frequency and phase correction using spectral correction; exponential apodization function (line broadening); fast Fourier transform; time averaging; frequency and phase correction based on fitting of the water and creatine (Cr) signals; and pairwise rejection of data for which fitting parameters were greater than three standard deviations from the mean; and subtraction to generate the edited difference spectrum (and extraction of the OFF spectrum). The output GABA and Glx concentrations were expressed in international units relative to water (GABA and Glx/water) and as an integral ratio relative to Cr (GABA/Cr and Glx/Cr). Voxels were co-registered for T1-weighted structural acquisition. The detailed process is shown in Fig. 1B. The spectra were visually examined for artifacts by two specialists (W.W. and X.Y.Z.). MRS data were excluded if they demonstrated significant motion artifacts or insufficient water suppression. Finally, data that met our quality criteria were included in the analysis.

### Statistical analysis

Normally distributed data are presented as the mean ± standard deviation. For continuous data comparisons among the three groups (healthy control, EM, and CM groups), a one-way analysis of variance and a post hoc analysis with the least significant difference method were performed. An independent samples t-test was applied to compare the two groups (EM and CM groups). Relationships between local GABA and/or Glx concentrations and headache characteristics were determined using Person’s correlation. The positive and negative correlation coefficients (r) represent positive and negative correlations. Statistical significance was set at *P* < 0.05. All statistical data were analyzed using SPSS software for Windows version 25.0 (SPSS Inc., Chicago, IL, USA).

## Results

### Demographics and clinical characteristics

**Fig. 2:**
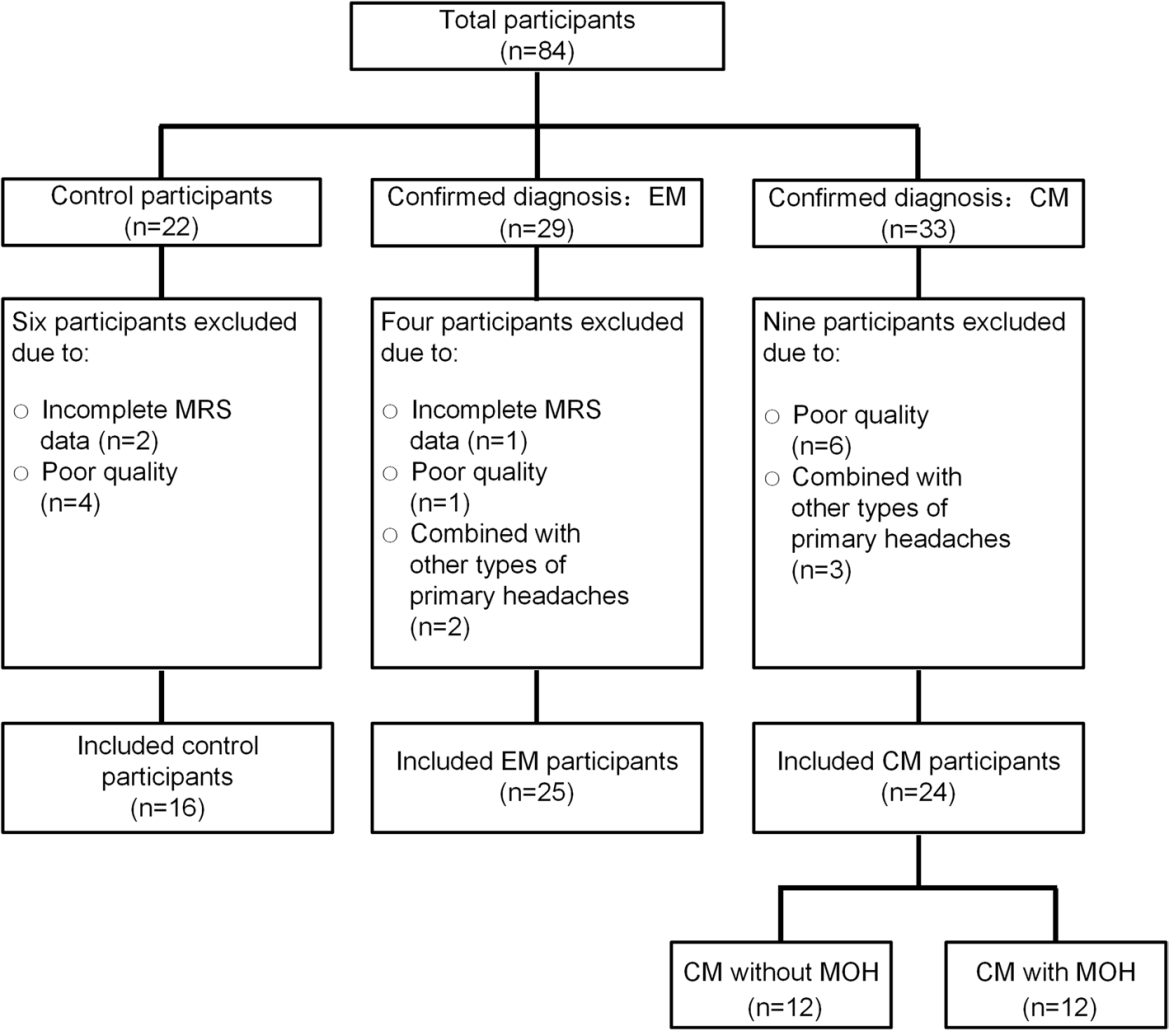
Flow of the participants during the study. Abbreviations: CM, chronic migraine; EM, episodic migraine; MOH, medication overuse headache; MRS, magnetic resonance spectroscopy

Sixteen control group participants, 25 EM group participants, and 24 CM group participants (12 with MOH and 12 without MOH) were included. Six control participants were excluded owe to incomplete MRS data (*n* = 2) and poor quality (*n* = 4). Four EM patients were excluded owe to incomplete MRS data (*n* = 1), poor quality (*n* = 1) and combined with other types of primary headaches (*n* = 2). Nine CM patients were excluded owe to poor quality data (*n* = 6) and combined with other types of primary headaches (*n* = 3) (Fig. 2). All participants were right-hand-dominant. The demographic and clinical characteristics of the different groups are shown in Tables 1 and 2. Compared to patients with EM, those with CM had more headache days (*p* < 0.001) and more severe disability, as assessed by the MIDAS (*p* = 0.033). Patients with CM and MOH had a longer disease duration (*p* = 0.034) and higher headache frequency (*p* = 0.018) than patients having CM without MOH. There were no significant differences in other characteristics of the groups.

**Table 1:** Comparisons of demographics and clinical characteristics between episodic and chronic migraine patients and normal controls

	**C (*n* = 16)**	**EM (*n* = 25)**	**CM (*n* = 24)**	***P***
Age, years	30.75 ± 5.98	37.16 ± 14.02	37.25 ± 15.15	0.236
Female, n (%)	8 (50.00)	19 (76.00)	16 (66.67)	0.237
Age at onset, years	NA	23.30 ± 9.78	20.17 ± 10.63	0.288
Disease duration, years	NA	14.74 ± 10.15	12.08 ± 9.07	0.340
Headache intensity^a^	NA	6.88 ± 1.92	7.25 ± 1.22	0.428
Headache frequency, days/month	NA	6.88 ± 3.56	21.88 ± 6.69	< 0.001***
BMI (kg/m^2^)	21.67 ± 2.72	22.51 ± 3.50	22.29 ± 3.59	0.564
MIDAs (0–270)	NA	52.60 ± 37.30	101.00 ± 63.16	0.033*
HIT-6 (36–78)	NA	68.40 ± 5.64	62.92 ± 9.80	0.131
PHQ-9 (0–27)	NA	4.60 ± 3.72	8.23 ± 5.56	0.090
GAD-7 (0–21)	NA	5.10 ± 5.43	6.31 ± 5.50	0.605
PSQI (0–21)	NA	9.50 ± 4.58	7.92 ± 4.56	0.428
MoCA (0–30)	NA	27.78 ± 1.86	27.33 ± 2.06	0.616

*C* Control, *EM* Episodic migraine, *CM* Chronic migraine, *NA* Not applicable, *BMI* Body Mass Index, *MIDAs* Migraine Disability Assessment Scale, *HIT-6* Headache Impact Test-6, *PHQ-9* Patient Health Questionnaire-9, *GAD-7* Generalized Anxiety Disorder-7, *PSQI* Pittsburgh Sleep Quality Index, *MoCA* Montreal Cognitive Assessment

^*^P < 0.05, Statistically significant

^***^P < 0.001, Statistically significant

^a^Headache intensity in a 0–10 numerical rating scale

**Table 2:** Comparisons of demographics and clinical characteristics between CM with and without MOH

	**Without MOH (*n* = 12)**	**With MOH (*n* = 12)**	***P***
Age, years	32.25 ± 17.96	43.25 ± 8.89	0.054
Female, n (%)	6 (50.00)	10 (83.33)	0.091
Age at onset, years	19.25 ± 12.71	21.08 ± 8.52	0.682
Disease duration, years	12.08 ± 9.28	22.17 ± 12.41	0.034*
Headache intensity^a^	6.92 ± 1.24	7.58 ± 1.16	0.188
Headache frequency, days/month	18.75 ± 5.17	25.00 ± 6.74	0.018*
BMI (kg/m^2^)	22.09 ± 2.70	22.48 ± 4.42	0.798
MIDAs (0–270)	108.57 ± 73.51	92.17 ± 54.00	0.661
HIT-6 (36–78)	65.57 ± 6.24	59.83 ± 12.75	0.313
PHQ-9 (0–27)	9.14 ± 6.09	7.17 ± 5.19	0.546
GAD-7 (0–21)	7.29 ± 6.37	5.17 ± 4.58	0.512
PSQI (0–21)	5.86 ± 4.14	10.80 ± 3.70	0.059
MoCA (0–30)	27.57 ± 1.40	27.00 ± 2.92	0.658

*MO* Medication overuse, BMI Body Mass Index, *MIDAs* Migraine Disability Assessment Scale, *HIT-6* Headache Impact Test-6, *PHQ-9* Patient Health Questionnaire-9, *GAD-7* Generalized Anxiety Disorder-7, *PSQI* Pittsburgh Sleep Quality Index, *MoCA* Montreal Cognitive Assessment

^*^P < 0.05, Statistically significant;

^a^Headache intensity in a 0–10 numerical rating scale

### Brain neurochemical levels in the control, EM, and CM groups

**Fig. 3:**
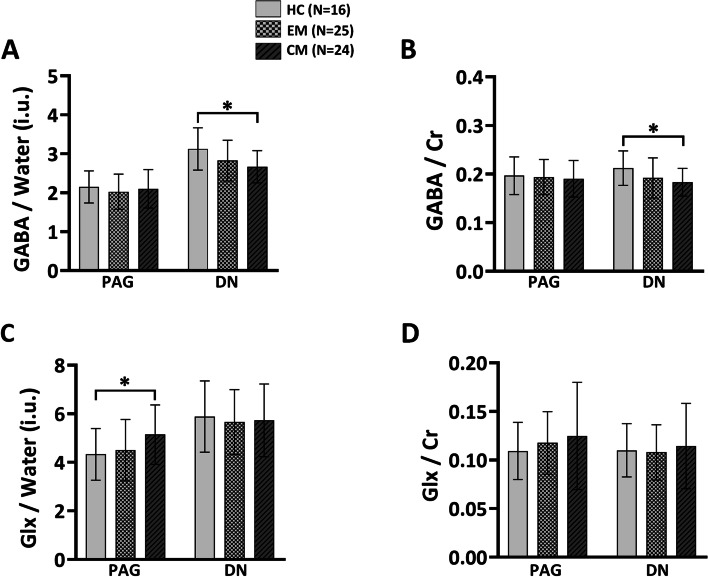
Brain neurochemical levels of the HC, EM, and CM groups. **a** GABA/water (international units [i.u.]) in the PAG and DN of the HC, EM, and CM groups. **b** GABA/Cr in the PAG and DN of the HC, EM, and CM groups. **c** Glx/water (i.u.) in the PAG and DN of the HC, EM, and CM groups. **d** Glx/Cr in the PAG and DN of the HC, EM, and CM groups. *P < 0.05 (statistically significant). Abbreviations: HC, healthy control; EM, episodic migraine; CM, chronic migraine; PAG, periaqueductal gray; DN, dentate nucleus; Cr, creatine; GABA, gamma-aminobutyric acid; Glx, glutamate/glutamine

The neurochemical levels in the PAG and DN of the three groups are shown in Fig. 3. Our results showed that the GABA/water and GABA/Cr levels were not significantly different in the PAG of the control, EM, and CM groups. However, the levels of GABA/water (*p* = 0.011) and GABA/Cr (*p* = 0.026) in the DN of the CM group were significantly lower than those of the control group. Our results also showed that Glx/water and Glx/Cr levels were not significantly different in the DN of the control, EM, and CM groups. However, the level of Glx/water (*p* = 0.049) in the PAG of the CM group was significantly higher than that of the control group. Patients with CM had a significantly higher Glx/Cr ratio in the PAG than the control group (*P* = 0.080).

### Brain neurochemical levels of patients having CM with and without MOH

**Fig. 4:**
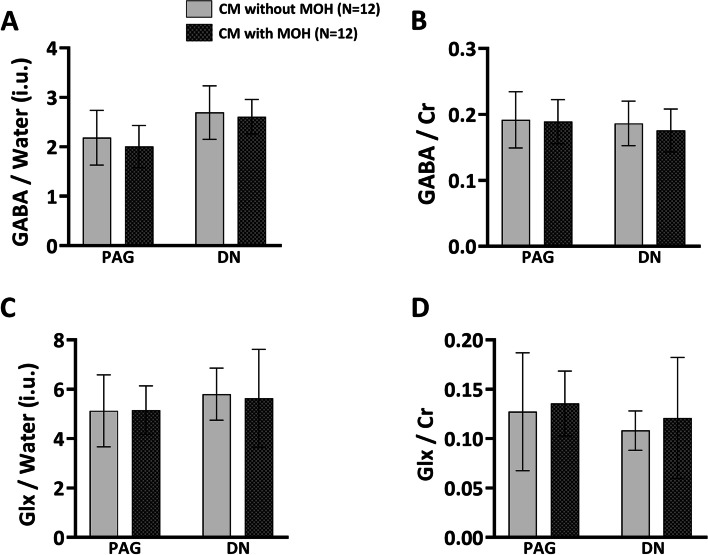
Brain neurochemical levels of the CM with and without MOH groups. **a** GABA/water (international units [i.u.]) in the PAG and DN of the patients having CM with and without MOH. **b** GABA/Cr in the PAG and DN of the patients having CM with and without MOH. **c** Glx/water (i.u.) in the PAG and DN of the patients having CM with and without MOH. **d** Glx/Cr in the PAG and DN of the patients having CM with and without MOH. Abbreviations: CM, chronic migraine; MOH, medication overuse headache; PAG, periaqueductal gray; DN, dentate nucleus; Cr, creatine; GABA, gamma-aminobutyric acid; Glx, glutamate/glutamine

The neurochemical levels in the PAG and DN of the three groups are shown in Fig. 4. Our results showed that GABA/water and GABA/Cr levels were not significantly different in the PAG and DN of the patients having CM with and without MOH. Our results also showed that Glx/water and Glx/Cr levels were not significantly different in the PAG and DN of patients having CM with and without MOH.

### Correlation between brain neurochemical levels and migraine characteristics

**Fig. 5:**
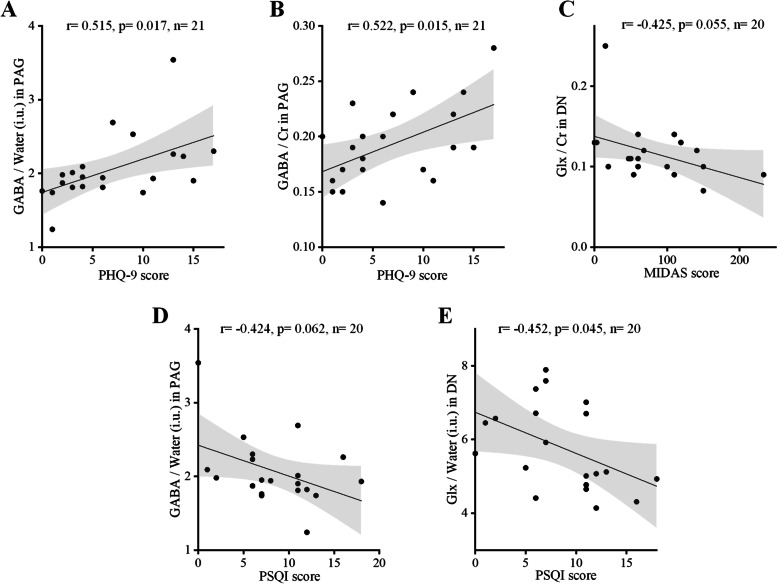
Associations between migraine characteristics and neurochemical levels. **a** Positive correlation between GABA/water in the PAG and the PHQ-9 score (*r* = 0.515, *p* = 0.017, *n* = 21). **b** Positive correlation between GABA/Cr in the PAG and the PHQ-9 score (*r* = 0.522, *p* = 0.015, *n* = 21). **c** Negative correlation between Glx/Cr in the DN and the MIDAS score (*r* = -0.425, *p* = 0.055, *n* = 20). **d** Negative correlation between GABA/water in the PAG and the PSQI score (*r* = -0.424, *p* = 0.062, *n* = 20). **E** Negative correlation between Glx/Water in the DN and the PSQI score (*r* = -0.452, *p* = 0.045, *n* = 20). Dots represent participants with migraine. The black regression line represents Pearson’s correlation coefficient (r). Gray shading represents the 95% confidence intervals of the partial correlations. Abbreviations: PAG, periaqueductal gray; DN, dentate nucleus; Cr, creatine; GABA, gamma-aminobutyric acid; Glx, glutamate/glutamine; MIDAS, migraine disability assessment scale; PHQ-9, Patient Health Questionnaire-9; PSQI, Pittsburgh Sleep Quality Index

During this study, we compared the correlation between migraine characteristics and different regional neurochemical levels of all patients with migraine (patients with EM and CM, *n* = 49). In patients with migraine, higher GABA levels (GABA/water: *r* = 0.515, *p* = 0.017, *n* = 21; GABA/Cr: *r* = 0.522, *p* = 0.015, *n* = 21) in the PAG were significantly associated with poorer sleep quality (higher PHQ-9 scores) (Fig. 5A, B). Lower Glx/Cr (*r* = -0.425, *p* = 0.055, *n* = 20) levels in the DN were associated with more severe migraine disability (Fig. 5C), and lower GABA/water (*r* = -0.424, *p* = 0.062, *n* = 20) and Glx/water (*r* = -0.452, *p* = 0.045, *n* = 20) levels were associated with poorer sleep quality (Fig. 5D, E); however, these associations were not significant. Additionally, we found that GABA and Glx levels in the PAG and DN of all patients were not associated with any other clinical characteristics (Additional file 1: Figs. S1-S8).

## Discussion

This study aimed to explore the pathogenesis of migraine chronification using the neurotransmitters GABA and Glx. We specifically investigated local GABA and Glx concentrations in the PAG and DN of the control, EM, and CM groups. Our results showed that GABA levels in the DN were significantly lower in the CM group than in the control group. We also found that Glx levels in the PAG were significantly higher in the CM group than in the control group. Additionally, we found that depression, disability, and sleep quality were strongly associated with neurochemical levels.

### DN and migraine

The cerebellum accounts for only 10% of the total volume of the brain, yet it contains more than 50% of the total number of neurons in the brain [24]. The cerebellum consists of two major parts, the cerebellar nucleus and the cortex. The cerebellar nuclei are the main output structures of the cerebellum and innervate several areas of the brainstem and forebrain [25]. The cerebellar cortex is divided, from the inside to the outside, into the granule cell layer, Purkinje cell layer, and molecular layer. Cerebellar nuclei neurons receive the majority of their inputs from GABAergic Purkinje cells that form the principal output neurons of the cerebellar cortex [26]. The DN is the largest cerebellar nucleus located lateral to the interposed nuclei. It receives input from the lateral hemisphere and cerebellar afferents that carry information from the cerebral cortex (via the pontine nuclei). It projects to the contralateral red nucleus and ventrolateral thalamic nucleus. Therefore, the DN has a pivotal role in cerebellar-related functions.

The cerebellum has canonically been implicated in various forms of motor control and coordination [27, 28]; however, recent evidence has suggested that it may have a role in regulating migraine [18]. Some MRI studies of migraine showed significantly increased cerebellar activity during the ictal phase compared to that during the interictal phase [29–31]. Increased cerebellar activity has been demonstrated in response to trigeminal noxious stimuli in patients with migraine and healthy subjects [32–35]. Several structural imaging studies have found ischemic cavities, subclinical infarcts, and lesions in the cerebellar cortex and white matter in patients with migraine, suggesting that the cerebellum is particularly vulnerable to atrophy and injury [36–41]. Some studies showed that the severity of cerebellum damage was associated with the frequency and duration of migraine attacks [42, 43]. Additionally, recent studies have reported altered cerebellar functional connectivity with migraine, suggesting that the cerebellum may be involved in pain regulation [29, 44].

Cerebellar connectivity with neuronal networks is the anatomical basis of functional regulation in migraine. As one of the key structures involved in migraine pathophysiology, the spinal trigeminal nucleus receives information from trigeminal ganglion cells innervating the meninges and cranial vasculature [45]. It has also been verified that nociceptive neurons in the spinal trigeminal nucleus directly project to the cerebellum and cerebellar areas, such as the inferior olive and pontine nuclei [46–49]. Additionally, the cerebellum has also been found to be reciprocally connected to the PAG [50, 51], and receive input from the locus coeruleus and parabrachial nucleus [52, 53], which are thought to have the capacity to modulate the activity of the trigeminal pathway. Studies of neurochemical levels of patients with migraine have provided additional evidence elucidating the pathological mechanisms. Other studies have also reported varying neurochemical levels in different brain regions of patients with migraine [15]. However, no studies of the role of GABA and Glx in the cerebellar DN with migraine have been reported. During this study, we found that the level of GABA in the DN of the CM group was significantly lower than that of the control group; however, there was no significant difference between the EM and control groups. This suggests that the decreased GABA level in the DN may weaken the inhibitory role of the cerebellum in pain regulation, thus participating in the chronic process of migraine.

### PAG and migraine

The PAG receives afferent information from nociceptive neurons and projects it to the thalamic nucleus, which is an important part of the ascending pain processing. The PAG also regulates pain sensations by projecting down to the rostroventromedial medulla and trigeminocervical complex [16]. The PAG is divided into four functional subdivisions, the dorsomedial PAG, dorsolateral PAG, lateral PAG, and ventrolateral PAG. Furthermore, within these separate subdivisions, there are differences in their responses to opioids and development of tolerance to analgesics and in the dual role of the ascending and descending pathways or the role of only the descending pathway. The PAG represents one of the most significant elements of the endogenous descending modulatory system and has attracted increasing attention regarding its role in migraine mechanisms. Several studies have reported structural changes in the PAG, such as increased volume, lesions, abnormal diffusion tensor imaging results, and iron deposition, in patients with migraine [54, 55]. Functional imaging has also indicated changes in hemodynamics and functional connectivity in the PAG of patients with migraine [54, 56, 57]. Few studies have examined the neurochemical metabolism of the PAG with EM and CM. Only Wang et al. reported the contents of N-acetylaspartate and choline in the PAG of healthy controls and patients with EM and CM; however, no significant differences were observed [58]. During this study, we found that the level of Glx in the PAG was significantly higher in patients with CM than in controls; however, this trend was not observed for patients with EM. Animal experiments have demonstrated that activation of glutamatergic neurons or inhibition of GABAergic neurons in the ventrolateral PAG can suppress nociception [59]. However, other functional areas of the PAG appear to have opposing effects [16]. Our MRS results showed the neurochemical level of the entire PAG region and did not distinguish between different functional regions. Therefore, we can conclude that an increase in the overall Glx level in the PAG is associated with CM. The disturbance of pain conduction and analgesia in the PAG may cause migraine chronification.

### MOH and neurochemical levels

The 1-year prevalence of MOH is 2 to 3% for the general population and at least 50% for individuals with CM overuse medication [60, 61]. The pathogenesis by which medication overuse facilitates migraine transformation is incompletely understood. Numerous studies have shown that MOH is associated with atypical structures and functions of brain regions responsible for pain processing and those that are commonly implicated in addiction [62]. Some hypotheses have been proposed, such as dysfunction of the descending antinociceptive network in the brainstem and disturbance of the serotonin system [63]. However, our results failed to show a difference in neurochemical levels of patients having CM with and without MOH, which is consistent with the conclusions of previous studies [58].

### Headache characteristics and neurochemical levels

Correlations between neurochemical levels and migraine characteristics remain unclear. Peek et al. demonstrated that improvements in migraine frequency, intensity, and disability are associated with increased GABA+ levels in the anterior cingulate cortex [64]. Bell et al. found that higher glutamate levels in the thalamus and higher GABA/Glx ratios in the sensorimotor cortex were associated with longer durations of pediatric migraine [10]. Additionally, lower GABA levels in the sensorimotor cortex are associated with more frequent migraine attacks [10]. However, our results regarding the DN and PAG did not show this trend. Our results showed that the increased level of GABA in the PAG was positively correlated with the degree of depression, and that the Glx level in the DN was negatively correlated with migraine-associated disability. Furthermore, we found that lower levels of GABA in the PAG and Glx in the DN represented poorer sleep quality. In summary, we believe that the neurochemical levels in the DN and PAG are involved in the regulation of depression and sleep and are closely related to disability severity.

### Limitations

There were some limitations in this study. First, the ROIs placed in the target regions could not be avoided the involvement of surrounding structures. Therefore, we could not be certain about the exact relevant substrate within the DN and PAG, but we believe the results could reflect the relative changes in these regions. Additionally, the resolution during scanning of the region of interest was limited; therefore, specific functional areas and nuclei could not be distinguished. Furthermore, as a single-center cross-sectional study, the results of the current study can’t provide the information about the changes of these neurochemical levels during a continuum of chronification progression and/or whether they are reversible. Multicenter studies with large study populations are required to validate the role of neurochemical levels in the migraine diagnosis.

## Conclusion

The pathogenesis of migraine chronification is not yet fully understood. Most relevant studies have focused on structural or functional imaging, omics, and immune inflammation; few have focused on the neurochemical level. MRS, as a noninvasive craniocerebral detection technique, provides a basis for obtaining the neurochemical level of the human brain. The findings of this study indicated that GABA and Glx levels in the DN and PAG may have a pertinent role in migraine chronification. These abnormal neurochemical levels may serve as potential markers of migraine chronification and provide new evidence regarding the monitoring and treatment of CM.

## Supplementary Information

**Additional file 1: Fig. S1.** Associations between headache frequency and neurochemical levels. (A) and (B) show the correlation between headache frequency and the GABA level in the PAG. (C) and (D) show the correlation between headache frequency and the Glx level in the PAG. (E) and (F) show the correlation between headache frequency and the GABA level in the DN. (G) and (H) show the correlation between headache frequency and the Glx level in the DN. Dots represent participants with migraine. The black regression line represents Pearson’s correlation coefficient (r). Gray shading represents the 95% confidence intervals of the partial correlations. PAG, periaqueductal gray; DN, dentate nucleus; GABA, gamma-aminobutyric acid; Glx, glutamate/glutamine. **Fig. S2.** Associations between disease duration and neurochemical levels. (A) and (B) show the correlation between disease duration and the GABA level in the PAG. (C) and (D) show the correlation between disease duration and the Glx level in the PAG. (E) and (F) show the correlation between disease duration and the GABA level in the DN. (G) and (H) show the correlation between disease duration and the Glx level in the DN. Dots represent participants with migraine. The black regression line represents Pearson’s correlation coefficient (r). Gray shading represents the 95% confidence intervals of the partial correlations. PAG, periaqueductal gray; DN, dentate nucleus; GABA, gamma-aminobutyric acid; Glx, glutamate/glutamine. **Fig. S3.** Associations between the MIDAS score and neurochemical levels. (A) and (B) show the correlation between the MIDAS score and GABA level in the PAG. (C) and (D) show the correlation between the MIDAS score and Glx level in the PAG. (E) and (F) show the correlation between the MIDAS score and GABA level in the DN. (G) and (H) show the correlation between the MIDAS score and Glx level in the DN. Dots represent participants with migraine. The black regression line represents Pearson’s correlation coefficient (r). Gray shading represents the 95% confidence intervals of the partial correlations. MIDAS, migraine disability assessment scale; PAG, periaqueductal gray; DN, dentate nucleus; GABA, gamma-aminobutyric acid; Glx, glutamate/glutamine. **Fig. S4.** Associations between the HIT-6 score and neurochemical levels. (A) and (B) show the correlation between the HIT-6 score and GABA level in the PAG. (C) and (D) show the correlation between the HIT-6 score and Glx level in the PAG. (E) and (F) show the correlation between the HIT-6 score and GABA level in the DN. (G) and (H) show the correlation between the HIT-6 score and Glx level in the DN. Dots represent participants with migraine. The black regression line represents Pearson’s correlation coefficient (r). Gray shading represents the 95% confidence intervals of the partial correlations. HIT-6, Headache Impact Test; PAG, periaqueductal gray; DN, dentate nucleus; GABA, gamma-aminobutyric acid; Glx, glutamate/glutamine. **Fig. S5.** Associations between the PHQ-9 score and neurochemical levels. (A) and (B) show the correlation between the PHQ-9 score and GABA level in the PAG. (C) and (D) show the correlation between the PHQ-9 score and Glx level in the PAG. (E) and (F) show the correlation between the PHQ-9 score and GABA level in the DN. (G) and (H) show the correlation between the PHQ-9 score and Glx level in the DN. Dots represent participants with migraine. The black regression line represents Pearson’s correlation coefficient (r). Gray shading represents the 95% confidence intervals of the partial correlations. Patient Health Questionnaire-9; PAG, periaqueductal gray; DN, dentate nucleus; GABA, gamma-aminobutyric acid; Glx, glutamate/glutamine. **Fig. S6.** Associations between the GAD-7 score and neurochemical levels. (A) and (B) show the correlation between the GAD-7 score and GABA level in the PAG. (C) and (D) show the correlation between the GAD-7 score and Glx level in the PAG. (E) and (F) show the correlation between the GAD-7 score and GABA level in the DN. (G) and (H) show the correlation between the GAD-7 score and Glx level in the DN. Dots represent participants with migraine. The black regression line represents Pearson’s correlation coefficient (r). Gray shading represents the 95% confidence intervals of the partial correlations. Generalized Anxiety Disorder-7; PAG, periaqueductal gray; DN, dentate nucleus; GABA, gamma-aminobutyric acid; Glx, glutamate/glutamine. **Fig. S7.** Associations between the PSQI score and neurochemical levels. (A) and (B) show the correlation between the PSQI score and GABA level in the PAG. (C) and (D) show the correlation between the PSQI score and Glx level in the PAG. (E) and (F) show the correlation between the PSQI score and GABA level in the DN. (G) and (H) show the correlation between the PSQI score and Glx level in the DN. Dots represent participants with migraine. The black regression line represents Pearson’s correlation coefficient (r). Gray shading represents the 95% confidence intervals of the partial correlations. PSQI, Pittsburgh Sleep Quality Index; PAG, periaqueductal gray; DN, dentate nucleus; GABA, gamma-aminobutyric acid; Glx, glutamate/glutamine. **Fig. S8.** Associations between the MoCA score and neurochemical levels. (A) and (B) show the correlation between the MoCA score and GABA level in the PAG. (C) and (D) show the correlation between the MoCA score and Glx level in the PAG. (E) and (F) show the correlation between the MoCA score and GABA level in the DN. (G) and (H) show the correlation between the MoCA score and Glx level in the DN. Dots represent participants with migraine. The black regression line represents Pearson’s correlation coefficient (r). Gray shading represents the 95% confidence intervals of the partial correlations. MoCA, Montreal Cognitive Assessment; PAG, periaqueductal gray; DN, dentate nucleus; GABA, gamma-aminobutyric acid; Glx, glutamate/glutamine**.**

## Data Availability

Data can be made available upon request.

## Abbreviations

CM: Chronic migraine
Cr: Creatine
DN: Dentate nucleus
EM: Episodic migraine
GABA: Gamma-aminobutyric acid
GAD-7: Generalized Anxiety Disorder-7
Glx: Glutamate and glutamine
ICHD-3: International Classification of Headache Diseases, 3rd edition
MIDAS: Migraine disability assessment scale
MoCA: Montreal Cognitive Assessment
MOH: Medication overuse headache
MRI: Magnetic resonance imaging
MRS: Magnetic resonance spectroscopy
PAG: Periaqueductal gray
PHQ-9: Patient Health Questionnaire-9
PSQI: Pittsburgh Sleep Quality Index

## References

[1] Stovner LJ, Hagen K, Linde M, Steiner TJ (2022). The global prevalence of headache: an update, with analysis of the influences of methodological factors on prevalence estimates. J Headache Pain.

[2] The International Classification of Headache Disorders (2013). 3rd edition (beta version). Cephalalgia.

[3] Filippi M, Messina R (2019). The chronic migraine brain: what have we learned from neuroimaging?. Front Neurol.

[4] Schwedt TJ (2014). Chronic migraine. BMJ (Clinical research ed) ed).

[5] Andreou AP, Edvinsson L (2019). Mechanisms of migraine as a chronic evolutive condition. J Headache Pain.

[6] Quintas M, Neto JL, Pereira-Monteiro J, Barros J, Sequeiros J, Sousa A, Alonso I, Lemos C (2013). Interaction between γ-aminobutyric acid A receptor genes: new evidence in migraine susceptibility. PLoS One.

[7] Chen T, Murrell M, Fowdar J, Roy B, Grealy R, Griffiths LR (2012). Investigation of the role of the GABRG2 gene variant in migraine. J Neurol Sci.

[8] Plummer PN, Colson NJ, Lewohl JM, MacKay RK, Fernandez F, Haupt LM, Griffiths LR (2011). Significant differences in gene expression of GABA receptors in peripheral blood leukocytes of migraineurs. Gene.

[9] Zielman R, Wijnen JP, Webb A, Onderwater GLJ, Ronen I, Ferrari MD, Kan HE, Terwindt GM, Kruit MC (2017). Cortical glutamate in migraine. Brain : a journal of neurology.

[10] Bell T, Stokoe M, Khaira A, Webb M, Noel M, Amoozegar F, Harris AD (2021). GABA and glutamate in pediatric migraine. Pain.

[11] Aguila ME, Lagopoulos J, Leaver AM, Rebbeck T, Hübscher M, Brennan PC, Refshauge KM (2015). Elevated levels of GABA+ in migraine detected using (1) H-MRS. NMR Biomed.

[12] Bridge H, Stagg CJ, Near J, Lau CI, Zisner A, Cader MZ (2015). Altered neurochemical coupling in the occipital cortex in migraine with visual aura. Cephalalgia : Int J Headache.

[13] Bigal ME, Hetherington H, Pan J, Tsang A, Grosberg B, Avdievich N, Friedman B, Lipton RB (2008). Occipital levels of GABA are related to severe headaches in migraine. Neurology.

[14] Russo A, Silvestro M, Tessitore A, Tedeschi G (2018). Advances in migraine neuroimaging and clinical utility: from the MRI to the bedside. Expert Rev Neurother.

[15] Younis S, Hougaard A, Vestergaard MB, Larsson HBW, Ashina M (2017). Migraine and magnetic resonance spectroscopy: a systematic review. Curr Opin Neurol.

[16] Vila-Pueyo M, Hoffmann J, Romero-Reyes M, Akerman S (2019). Brain structure and function related to headache: Brainstem structure and function in headache. Cephalalgia : Int J Headache.

[17] Messina R, Filippi M, Goadsby PJ (2018). Recent advances in headache neuroimaging. Curr Opin Neurol.

[18] Kros L, Angueyra Aristizábal CA, Khodakhah K (2018). Cerebellar involvement in migraine. Cephalalgia.

[19] Headache Classification Committee of the International Headache Society (IHS) (2018). The international classification of headache disorders, 3rd edition. Cephalalgia.

[20] Vandenbussche N, Laterza D, Lisicki M, Lloyd J, Lupi C, Tischler H, Toom K, Vandervorst F, Quintana S, Paemeleire K (2018). Medication-overuse headache: a widely recognized entity amidst ongoing debate. J Headache Pain.

[21] Takahashi TT, Ornello R, Quatrosi G, Torrente A, Albanese M, Vigneri S, Guglielmetti M, De MariaMarco C, Dutordoir C, Colangeli E (2021). Medication overuse and drug addiction: a narrative review from addiction perspective. J Headache Pain.

[22] ParejaRomán J, Jiménez Hernández MD, MaestuUnturbe C, Ramírez-Castillejo MdC (2020). Chronic Migraine with Medication Overuse: Clinical Pattern and Evolution from a Retrospective Cohort in Seville, Spain. SN Comprehensive Clin Med.

[23] Edden RA, Puts NA, Harris AD, Barker PB, Evans CJ (2014). Gannet: a batch-processing tool for the quantitative analysis of gamma-aminobutyric acid–edited MR spectroscopy spectra. JMRI.

[24] D'Angelo E (2019). The cerebellum gets social. Science (New York, NY).

[25] Palesi F, Tournier JD, Calamante F, Muhlert N, Castellazzi G, Chard D, D'Angelo E, Wheeler-Kingshott CA (2015). Contralateral cerebello-thalamo-cortical pathways with prominent involvement of associative areas in humans in vivo. Brain Struct Funct.

[26] De Zeeuw CI, Berrebi AS (1995). Postsynaptic targets of Purkinje cell terminals in the cerebellar and vestibular nuclei of the rat. Eur J Neurosci.

[27] Holmes G (1939). THE CEREBELLUM OF MAN1. Brain : J Neurol.

[28] Eccles JC (1969). The development of the cerebellum of vertebrates in relation to the control of movement. Naturwissenschaften.

[29] Mehnert J, May A (2019). Functional and structural alterations in the migraine cerebellum. J Cereb Blood Flow Metab.

[30] Stankewitz A, May A (2011). Increased limbic and brainstem activity during migraine attacks following olfactory stimulation. Neurology.

[31] Shin JH, Kim YK, Kim HJ, Kim JS (2014). Altered brain metabolism in vestibular migraine: comparison of interictal and ictal findings. Cephalalgia.

[32] Russo A, Tessitore A, Esposito F, Marcuccio L, Giordano A, Conforti R, Truini A, Paccone A, d'Onofrio F, Tedeschi G (2012). Pain processing in patients with migraine: an event-related fMRI study during trigeminal nociceptive stimulation. J Neurol.

[33] Moulton EA, Becerra L, Maleki N, Pendse G, Tully S, Hargreaves R, Burstein R, Borsook D (2011). Painful heat reveals hyperexcitability of the temporal pole in interictal and ictal migraine States. Cereb Cortex.

[34] Schwedt TJ, Chong CD, Chiang CC, Baxter L, Schlaggar BL, Dodick DW (2014). Enhanced pain-induced activity of pain-processing regions in a case-control study of episodic migraine. Cephalalgia.

[35] Stankewitz A, Voit HL, Bingel U, Peschke C, May A (2010). A new trigemino-nociceptive stimulation model for event-related fMRI. Cephalalgia.

[36] Kruit MC, Launer LJ, Ferrari MD, van Buchem MA (2005). Infarcts in the posterior circulation territory in migraine. The population-based MRI CAMERA study. Brain : J Neurol.

[37] Zaidat OO (2004). Migraine as a risk factor for subclinical brain lesions. Jama.

[38] Laurell K, Artto V, Bendtsen L, Hagen K, Kallela M, Meyer EL, Putaala J, Tronvik E, Zwart JA, Linde M (2011). Migrainous infarction: a Nordic multicenter study. Eur J Neurol.

[39] Scher AI, Gudmundsson LS, Sigurdsson S, Ghambaryan A, Aspelund T, Eiriksdottir G, van Buchem MA, Gudnason V, Launer LJ (2009). Migraine headache in middle age and late-life brain infarcts. JAMA.

[40] Messina R, Rocca MA, Colombo B, Teggi R, Falini A, Comi G, Filippi M (2017). Structural brain abnormalities in patients with vestibular migraine. J Neurol.

[41] Qin Z, He XW, Zhang J, Xu S, Li GF, Su J, Shi YH, Ban S, Hu Y, Liu YS (2019). Structural changes of cerebellum and brainstem in migraine without aura. J Headache Pain.

[42] Schmitz N, Admiraal-Behloul F, Arkink EB, Kruit MC, Schoonman GG, Ferrari MD, van Buchem MA (2008). Attack frequency and disease duration as indicators for brain damage in migraine. Headache.

[43] Ruscheweyh R, Kühnel M, Filippopulos F, Blum B, Eggert T, Straube A (2014). Altered experimental pain perception after cerebellar infarction. Pain.

[44] Liu HY, Lee PL, Chou KH, Lai KL, Wang YF, Chen SP, Chen WT, Wang SJ (2020). The cerebellum is associated with 2-year prognosis in patients with high-frequency migraine. J Headache Pain.

[45] Burstein R, Noseda R, Borsook D (2015). Migraine: multiple processes, complex pathophysiology. J Neurosci.

[46] Carpenter MB, Hanna GR (1961). Fiber projections from the spinal trigeminal nucleus in the cat. J Comp Neurol.

[47] Hayashi H, Sumino R, Sessle BJ (1984). Functional organization of trigeminal subnucleus interpolaris: nociceptive and innocuous afferent inputs, projections to thalamus, cerebellum, and spinal cord, and descending modulation from periaqueductal gray. J Neurophysiol.

[48] Huerta MF, Frankfurter A, Harting JK (1983). Studies of the principal sensory and spinal trigeminal nuclei of the rat: projections to the superior colliculus, inferior olive, and cerebellum. J Comp Neurol.

[49] Bukowska D, Mierzejewska-Krzyzowska B, Zguczyński L (2006). Topography and axonal collaterals of trigeminocerebellar projection to the paramedian lobule and uvula in the rabbit cerebellum. Acta Neurobiol Exp.

[50] Teune TM, van der Burg J, van der Moer J, Voogd J, Ruigrok TJ (2000). Topography of cerebellar nuclear projections to the brain stem in the rat. Prog Brain Res.

[51] Sillery E, Bittar RG, Robson MD, Behrens TE, Stein J, Aziz TZ, Johansen-Berg H (2005). Connectivity of the human periventricular-periaqueductal gray region. J Neurosurg.

[52] Dietrichs E (1985). Divergent axon collaterals to cerebellum and amygdala from neurons in the parabrachial nucleus, the nucleus locus coeruleus and some adjacent nuclei. A fluorescent double labelling study using rhodamine labelled latex microspheres and fast blue as retrograde tracers. Anat Embryol (Berl).

[53] Dietrichs E (1988). Cerebellar cortical and nuclear afferents from the feline locus coeruleus complex. Neuroscience.

[54] Linnman C, Moulton EA, Barmettler G, Becerra L, Borsook D (2012). Neuroimaging of the periaqueductal gray: state of the field. Neuroimage.

[55] Domínguez C, López A, Ramos-Cabrer P, Vieites-Prado A, Pérez-Mato M, Villalba C, Sobrino T, Rodriguez-Osorio X, Campos F, Castillo J (2019). Iron deposition in periaqueductal gray matter as a potential biomarker for chronic migraine. Neurology.

[56] Schwedt TJ, Chiang CC, Chong CD, Dodick DW (2015). Functional MRI of migraine. The Lancet Neurology.

[57] Linnman C, Beucke JC, Jensen KB, Gollub RL, Kong J (2012). Sex similarities and differences in pain-related periaqueductal gray connectivity. Pain.

[58] Lai TH, Fuh JL, Lirng JF, Lin CP, Wang SJ (2012). Brainstem 1H-MR spectroscopy in episodic and chronic migraine. J Headache Pain.

[59] Samineni VK, Grajales-Reyes JG, Copits BA, O'Brien DE, Trigg SL, Gomez AM, Bruchas MR, Gereau RWt (2017) Divergent Modulation of Nociception by Glutamatergic and GABAergic Neuronal Subpopulations in the Periaqueductal Gray. eNeuro 4(2):1–13 10.1523/ENEURO.0129-16.2017 PMC5370278 28374016

[60] Steiner TJ, Stovner LJ, Katsarava Z, Lainez JM, Lampl C, Lantéri-Minet M, Rastenyte D, de la RuizTorre E, Tassorelli C, Barré J (2014). The impact of headache in Europe: principal results of the Eurolight project. J Headache Pain.

[61] Westergaard ML, Glümer C, Hansen EH, Jensen RH (2014). Prevalence of chronic headache with and without medication overuse: associations with socioeconomic position and physical and mental health status. Pain.

[62] Schwedt TJ, Chong CD (2017). Medication overuse headache: pathophysiological insights from structural and functional brain MRI research. Headache.

[63] Cupini LM, Calabresi P (2005). Medication-overuse headache: pathophysiological insights. J Headache Pain.

[64] Peek AL, Leaver AM, Foster S, Puts NA, Oeltzschner G, Henderson L, Galloway G, Ng K, Refshauge K, Rebbeck T (2021). Increase in ACC GABA+ levels correlate with decrease in migraine frequency, intensity and disability over time. J Headache Pain.

