# Supplementary figures and images for: Gamma-aminobutyric acid and glutamate/glutamine levels in the dentate nucleus and periaqueductal gray with episodic and chronic migraine: a proton magnetic resonance spectroscopy study
Source: J Headache Pain. 2022 Jul 15;23(1):83. doi: 10.1186/s10194-022-01452-6 (PMC9287958; doi:10.1186/s10194-022-01452-6)

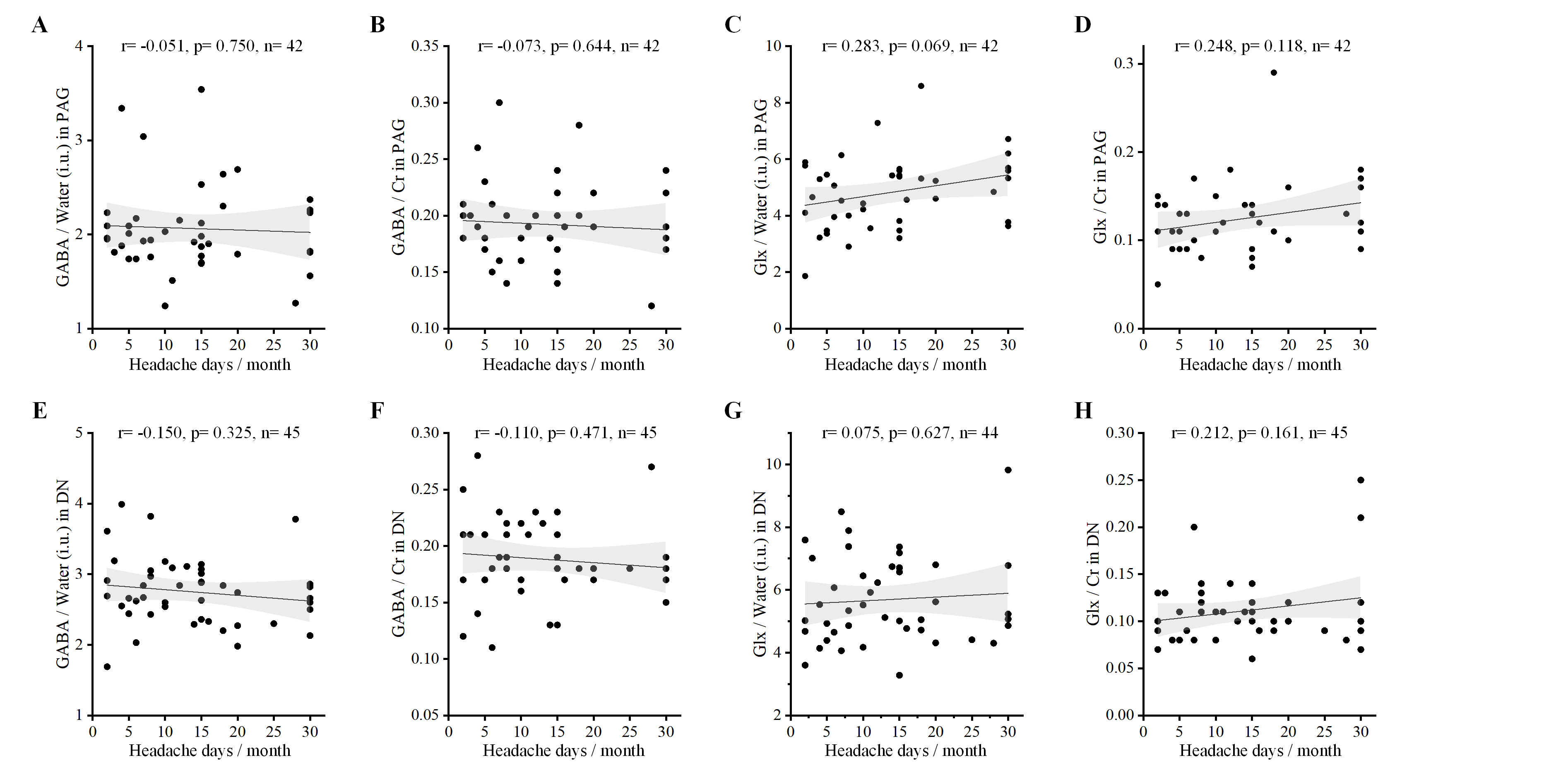

Supplement: Supplementary file 1 — Additional file 1: Fig. S1. Associations between headache frequency and neurochemical levels. (A) and (B) show the correlation between headache frequency and the GABA level in the PAG. (C) and (D) show the correlation between headache frequency and the Glx level in the PAG. (E) and (F) show the correlation between headache frequency and the GABA level in the DN. (G) and (H) show the correlation between headache frequency and the Glx level in the DN. Dots represent participants with migraine. The black regression line represents Pearson’s correlation coefficient (r). Gray shading represents the 95% confidence intervals of the partial correlations. PAG, periaqueductal gray; DN, dentate nucleus; GABA, gamma-aminobutyric acid; Glx, glutamate/glutamine. Fig. S2. Associations between disease duration and neurochemical levels. (A) and (B) show the correlation between disease duration and the GABA level in the PAG. (C) and (D) show the correlation between disease duration and the Glx level in the PAG. (E) and (F) show the correlation between disease duration and the GABA level in the DN. (G) and (H) show the correlation between disease duration and the Glx level in the DN. Dots represent participants with migraine. The black regression line represents Pearson’s correlation coefficient (r). Gray shading represents the 95% confidence intervals of the partial correlations. PAG, periaqueductal gray; DN, dentate nucleus; GABA, gamma-aminobutyric acid; Glx, glutamate/glutamine. Fig. S3. Associations between the MIDAS score and neurochemical levels. (A) and (B) show the correlation between the MIDAS score and GABA level in the PAG. (C) and (D) show the correlation between the MIDAS score and Glx level in the PAG. (E) and (F) show the correlation between the MIDAS score and GABA level in the DN. (G) and (H) show the correlation between the MIDAS score and Glx level in the DN. Dots represent participants with migraine. The black regression line represents Pearson’s correlation coeff [file 10194_2022_1452_MOESM1_ESM.zip › FigureS1.tif]

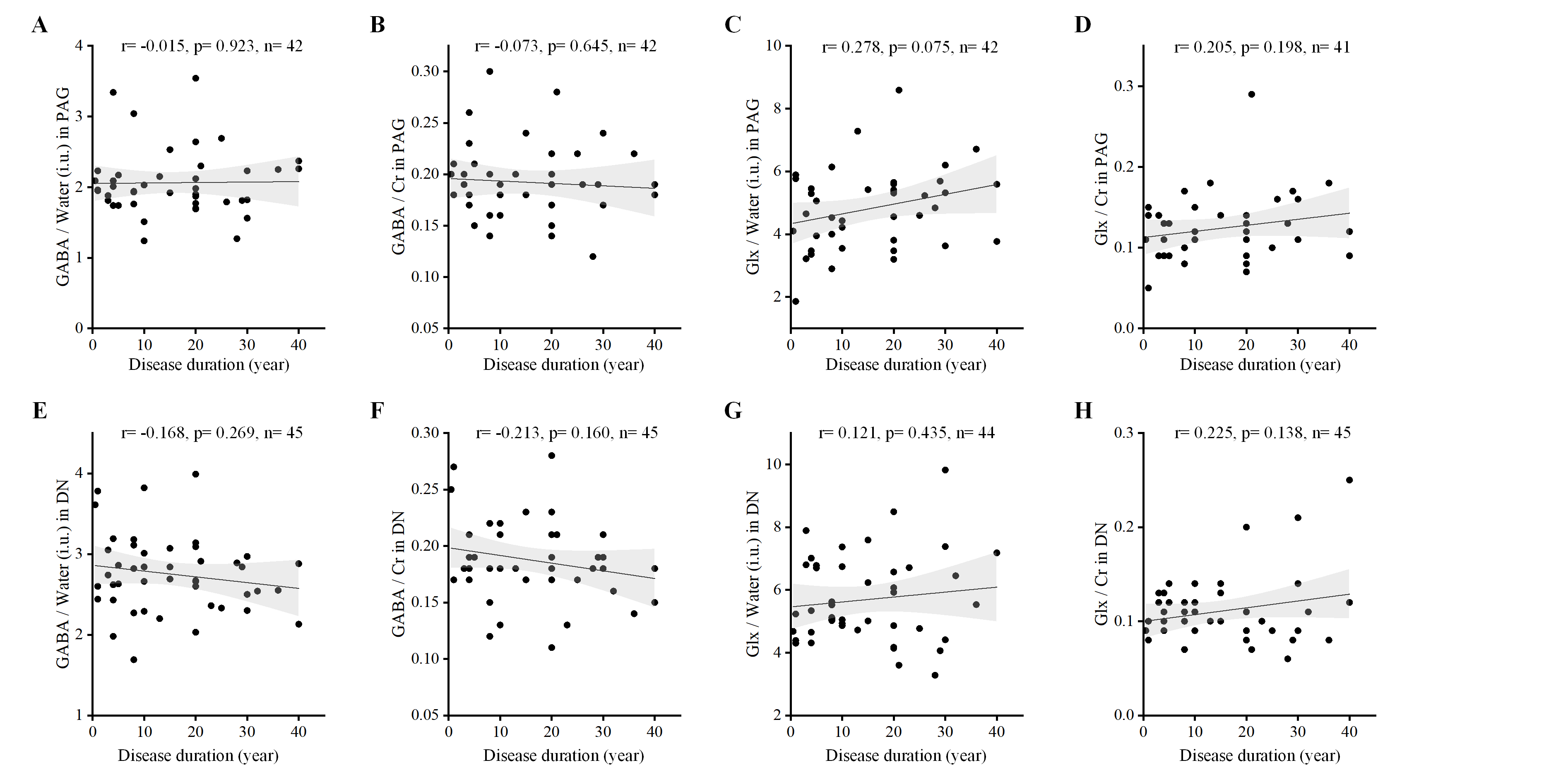

Supplement: Supplementary file 1 — Additional file 1: Fig. S1. Associations between headache frequency and neurochemical levels. (A) and (B) show the correlation between headache frequency and the GABA level in the PAG. (C) and (D) show the correlation between headache frequency and the Glx level in the PAG. (E) and (F) show the correlation between headache frequency and the GABA level in the DN. (G) and (H) show the correlation between headache frequency and the Glx level in the DN. Dots represent participants with migraine. The black regression line represents Pearson’s correlation coefficient (r). Gray shading represents the 95% confidence intervals of the partial correlations. PAG, periaqueductal gray; DN, dentate nucleus; GABA, gamma-aminobutyric acid; Glx, glutamate/glutamine. Fig. S2. Associations between disease duration and neurochemical levels. (A) and (B) show the correlation between disease duration and the GABA level in the PAG. (C) and (D) show the correlation between disease duration and the Glx level in the PAG. (E) and (F) show the correlation between disease duration and the GABA level in the DN. (G) and (H) show the correlation between disease duration and the Glx level in the DN. Dots represent participants with migraine. The black regression line represents Pearson’s correlation coefficient (r). Gray shading represents the 95% confidence intervals of the partial correlations. PAG, periaqueductal gray; DN, dentate nucleus; GABA, gamma-aminobutyric acid; Glx, glutamate/glutamine. Fig. S3. Associations between the MIDAS score and neurochemical levels. (A) and (B) show the correlation between the MIDAS score and GABA level in the PAG. (C) and (D) show the correlation between the MIDAS score and Glx level in the PAG. (E) and (F) show the correlation between the MIDAS score and GABA level in the DN. (G) and (H) show the correlation between the MIDAS score and Glx level in the DN. Dots represent participants with migraine. The black regression line represents Pearson’s correlation coeff [file 10194_2022_1452_MOESM1_ESM.zip › FigureS2.tif]

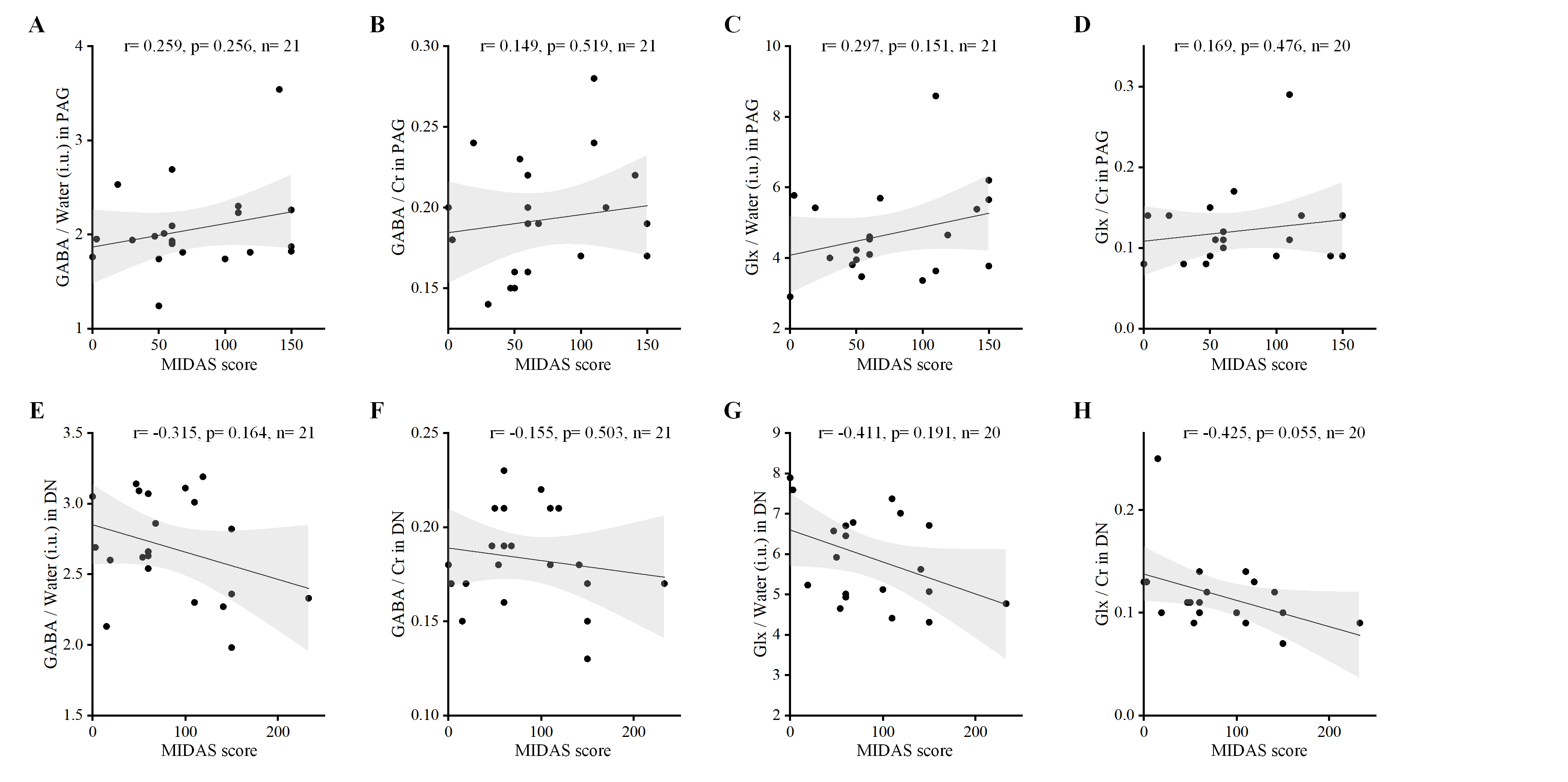

Supplement: Supplementary file 1 — Additional file 1: Fig. S1. Associations between headache frequency and neurochemical levels. (A) and (B) show the correlation between headache frequency and the GABA level in the PAG. (C) and (D) show the correlation between headache frequency and the Glx level in the PAG. (E) and (F) show the correlation between headache frequency and the GABA level in the DN. (G) and (H) show the correlation between headache frequency and the Glx level in the DN. Dots represent participants with migraine. The black regression line represents Pearson’s correlation coefficient (r). Gray shading represents the 95% confidence intervals of the partial correlations. PAG, periaqueductal gray; DN, dentate nucleus; GABA, gamma-aminobutyric acid; Glx, glutamate/glutamine. Fig. S2. Associations between disease duration and neurochemical levels. (A) and (B) show the correlation between disease duration and the GABA level in the PAG. (C) and (D) show the correlation between disease duration and the Glx level in the PAG. (E) and (F) show the correlation between disease duration and the GABA level in the DN. (G) and (H) show the correlation between disease duration and the Glx level in the DN. Dots represent participants with migraine. The black regression line represents Pearson’s correlation coefficient (r). Gray shading represents the 95% confidence intervals of the partial correlations. PAG, periaqueductal gray; DN, dentate nucleus; GABA, gamma-aminobutyric acid; Glx, glutamate/glutamine. Fig. S3. Associations between the MIDAS score and neurochemical levels. (A) and (B) show the correlation between the MIDAS score and GABA level in the PAG. (C) and (D) show the correlation between the MIDAS score and Glx level in the PAG. (E) and (F) show the correlation between the MIDAS score and GABA level in the DN. (G) and (H) show the correlation between the MIDAS score and Glx level in the DN. Dots represent participants with migraine. The black regression line represents Pearson’s correlation coeff [file 10194_2022_1452_MOESM1_ESM.zip › FigureS3.tif]

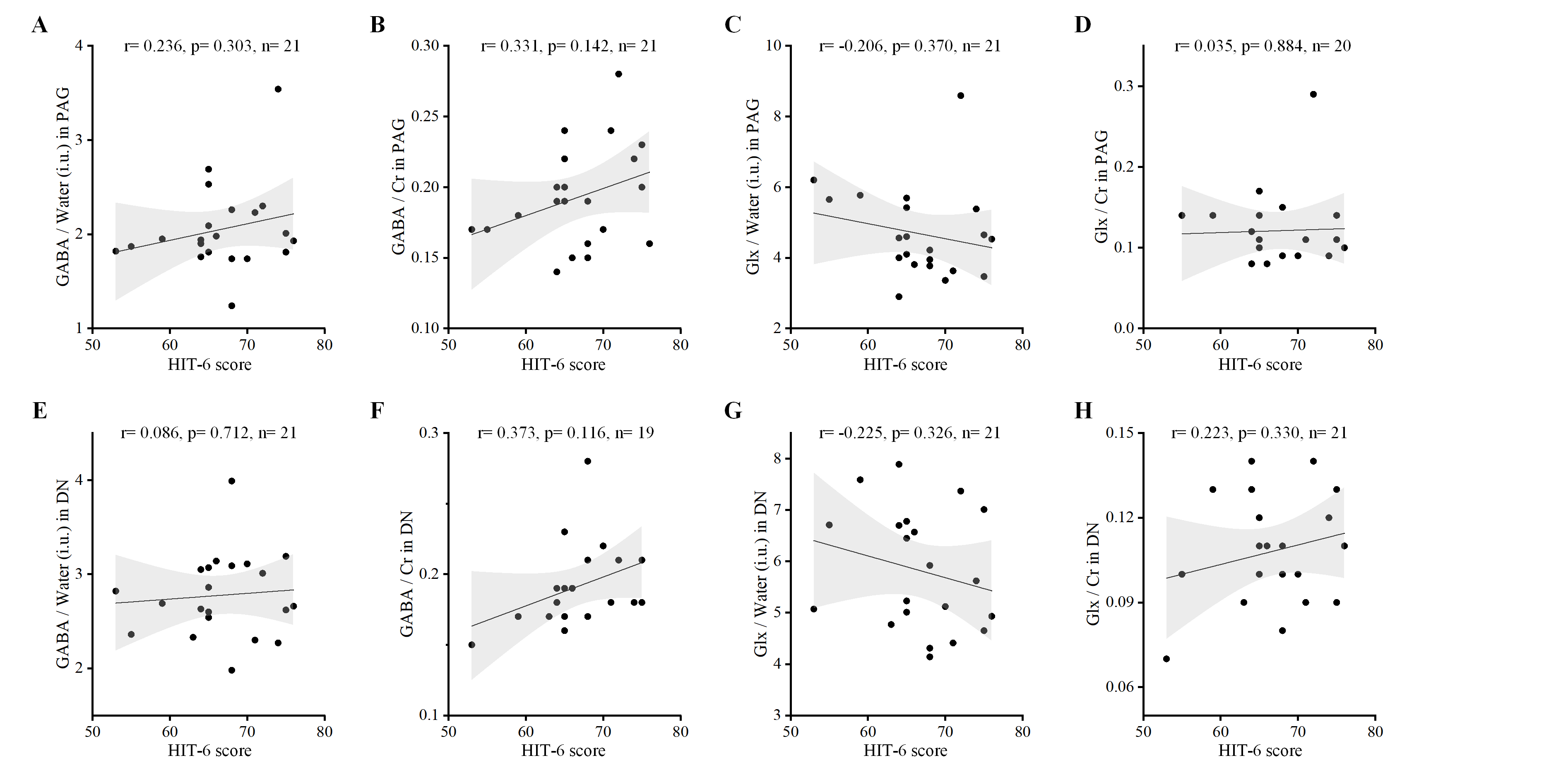

Supplement: Supplementary file 1 — Additional file 1: Fig. S1. Associations between headache frequency and neurochemical levels. (A) and (B) show the correlation between headache frequency and the GABA level in the PAG. (C) and (D) show the correlation between headache frequency and the Glx level in the PAG. (E) and (F) show the correlation between headache frequency and the GABA level in the DN. (G) and (H) show the correlation between headache frequency and the Glx level in the DN. Dots represent participants with migraine. The black regression line represents Pearson’s correlation coefficient (r). Gray shading represents the 95% confidence intervals of the partial correlations. PAG, periaqueductal gray; DN, dentate nucleus; GABA, gamma-aminobutyric acid; Glx, glutamate/glutamine. Fig. S2. Associations between disease duration and neurochemical levels. (A) and (B) show the correlation between disease duration and the GABA level in the PAG. (C) and (D) show the correlation between disease duration and the Glx level in the PAG. (E) and (F) show the correlation between disease duration and the GABA level in the DN. (G) and (H) show the correlation between disease duration and the Glx level in the DN. Dots represent participants with migraine. The black regression line represents Pearson’s correlation coefficient (r). Gray shading represents the 95% confidence intervals of the partial correlations. PAG, periaqueductal gray; DN, dentate nucleus; GABA, gamma-aminobutyric acid; Glx, glutamate/glutamine. Fig. S3. Associations between the MIDAS score and neurochemical levels. (A) and (B) show the correlation between the MIDAS score and GABA level in the PAG. (C) and (D) show the correlation between the MIDAS score and Glx level in the PAG. (E) and (F) show the correlation between the MIDAS score and GABA level in the DN. (G) and (H) show the correlation between the MIDAS score and Glx level in the DN. Dots represent participants with migraine. The black regression line represents Pearson’s correlation coeff [file 10194_2022_1452_MOESM1_ESM.zip › FigureS4.tif]

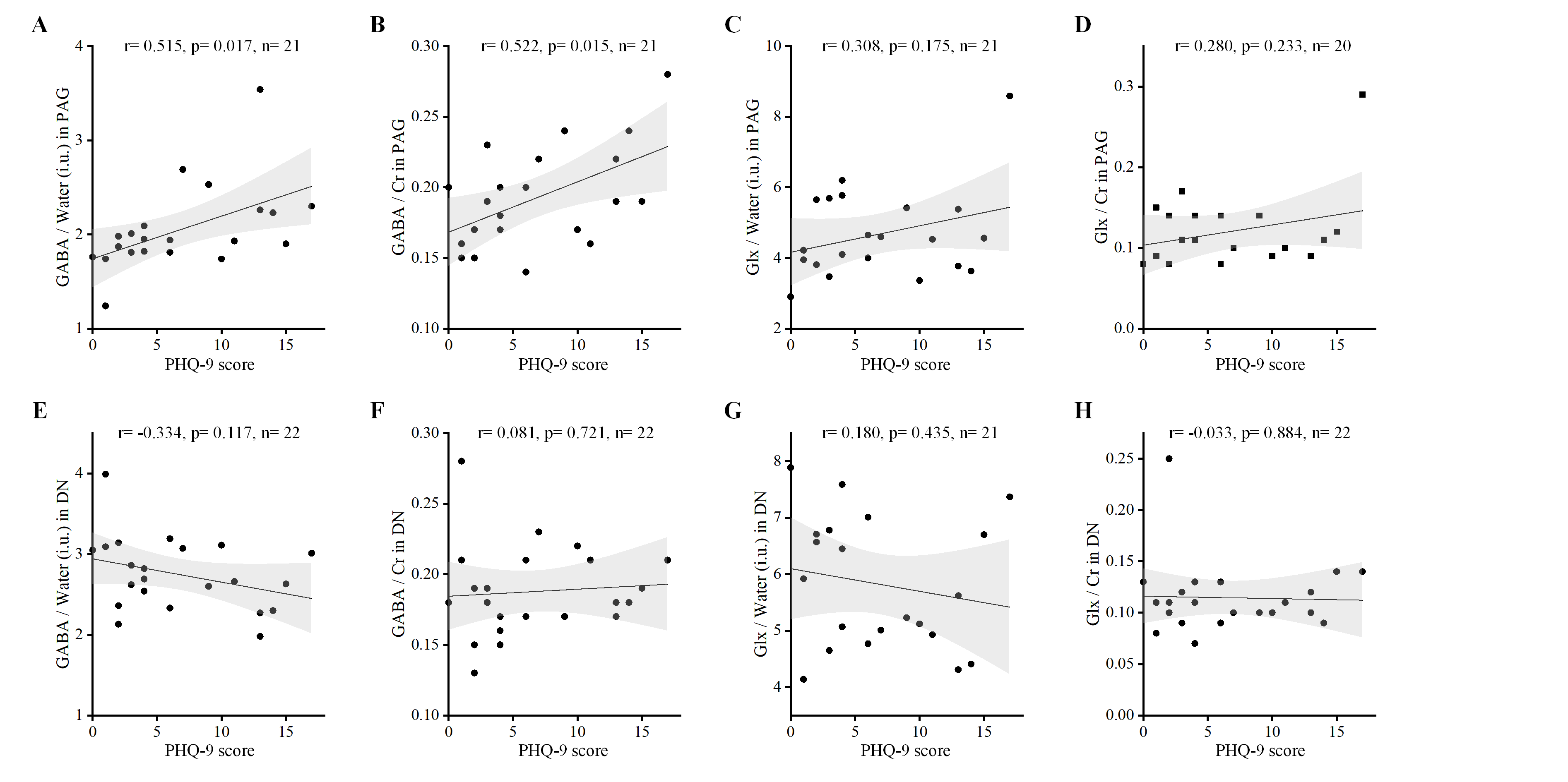

Supplement: Supplementary file 1 — Additional file 1: Fig. S1. Associations between headache frequency and neurochemical levels. (A) and (B) show the correlation between headache frequency and the GABA level in the PAG. (C) and (D) show the correlation between headache frequency and the Glx level in the PAG. (E) and (F) show the correlation between headache frequency and the GABA level in the DN. (G) and (H) show the correlation between headache frequency and the Glx level in the DN. Dots represent participants with migraine. The black regression line represents Pearson’s correlation coefficient (r). Gray shading represents the 95% confidence intervals of the partial correlations. PAG, periaqueductal gray; DN, dentate nucleus; GABA, gamma-aminobutyric acid; Glx, glutamate/glutamine. Fig. S2. Associations between disease duration and neurochemical levels. (A) and (B) show the correlation between disease duration and the GABA level in the PAG. (C) and (D) show the correlation between disease duration and the Glx level in the PAG. (E) and (F) show the correlation between disease duration and the GABA level in the DN. (G) and (H) show the correlation between disease duration and the Glx level in the DN. Dots represent participants with migraine. The black regression line represents Pearson’s correlation coefficient (r). Gray shading represents the 95% confidence intervals of the partial correlations. PAG, periaqueductal gray; DN, dentate nucleus; GABA, gamma-aminobutyric acid; Glx, glutamate/glutamine. Fig. S3. Associations between the MIDAS score and neurochemical levels. (A) and (B) show the correlation between the MIDAS score and GABA level in the PAG. (C) and (D) show the correlation between the MIDAS score and Glx level in the PAG. (E) and (F) show the correlation between the MIDAS score and GABA level in the DN. (G) and (H) show the correlation between the MIDAS score and Glx level in the DN. Dots represent participants with migraine. The black regression line represents Pearson’s correlation coeff [file 10194_2022_1452_MOESM1_ESM.zip › FigureS5.tif]

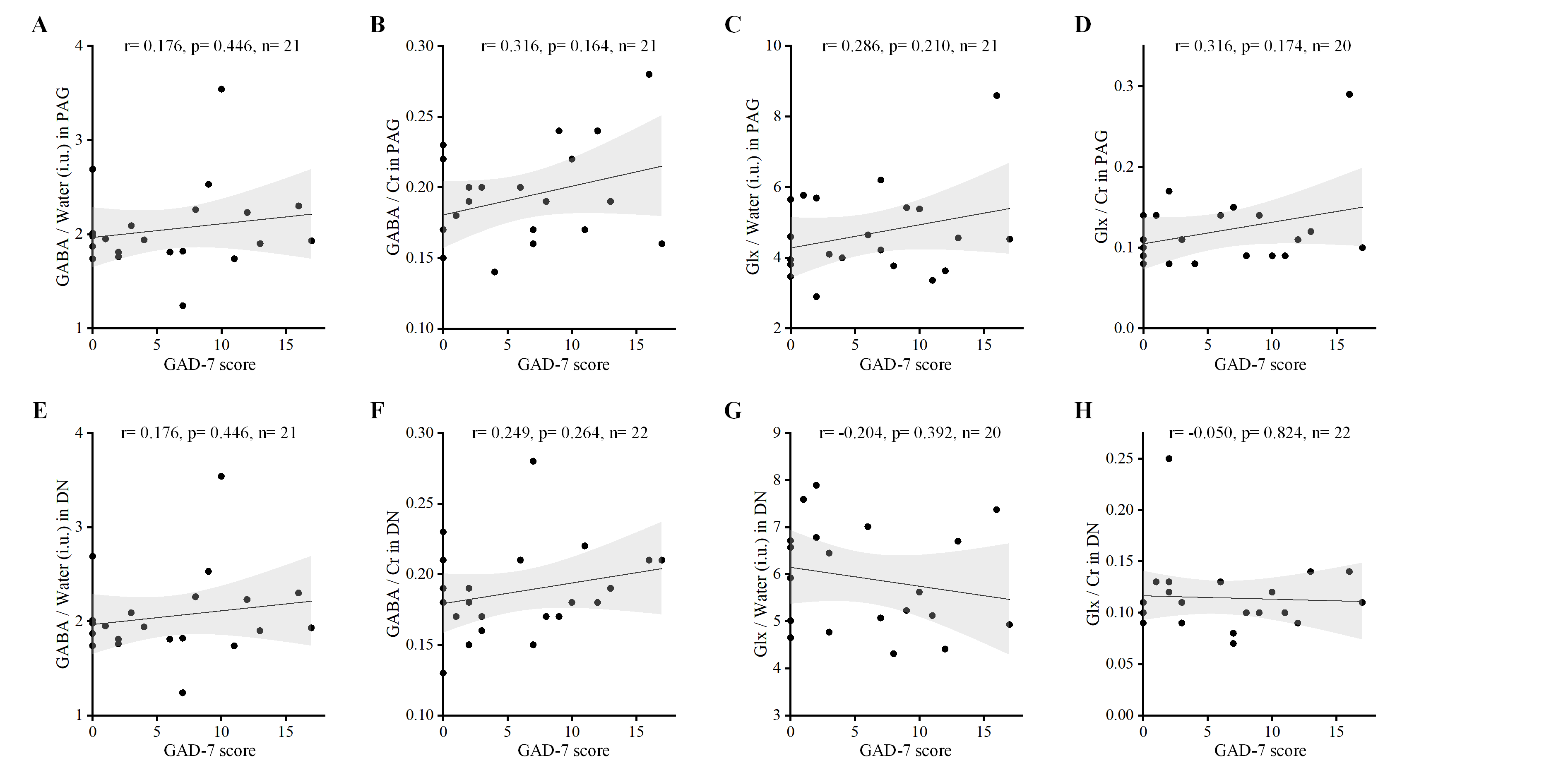

Supplement: Supplementary file 1 — Additional file 1: Fig. S1. Associations between headache frequency and neurochemical levels. (A) and (B) show the correlation between headache frequency and the GABA level in the PAG. (C) and (D) show the correlation between headache frequency and the Glx level in the PAG. (E) and (F) show the correlation between headache frequency and the GABA level in the DN. (G) and (H) show the correlation between headache frequency and the Glx level in the DN. Dots represent participants with migraine. The black regression line represents Pearson’s correlation coefficient (r). Gray shading represents the 95% confidence intervals of the partial correlations. PAG, periaqueductal gray; DN, dentate nucleus; GABA, gamma-aminobutyric acid; Glx, glutamate/glutamine. Fig. S2. Associations between disease duration and neurochemical levels. (A) and (B) show the correlation between disease duration and the GABA level in the PAG. (C) and (D) show the correlation between disease duration and the Glx level in the PAG. (E) and (F) show the correlation between disease duration and the GABA level in the DN. (G) and (H) show the correlation between disease duration and the Glx level in the DN. Dots represent participants with migraine. The black regression line represents Pearson’s correlation coefficient (r). Gray shading represents the 95% confidence intervals of the partial correlations. PAG, periaqueductal gray; DN, dentate nucleus; GABA, gamma-aminobutyric acid; Glx, glutamate/glutamine. Fig. S3. Associations between the MIDAS score and neurochemical levels. (A) and (B) show the correlation between the MIDAS score and GABA level in the PAG. (C) and (D) show the correlation between the MIDAS score and Glx level in the PAG. (E) and (F) show the correlation between the MIDAS score and GABA level in the DN. (G) and (H) show the correlation between the MIDAS score and Glx level in the DN. Dots represent participants with migraine. The black regression line represents Pearson’s correlation coeff [file 10194_2022_1452_MOESM1_ESM.zip › FigureS6.tif]

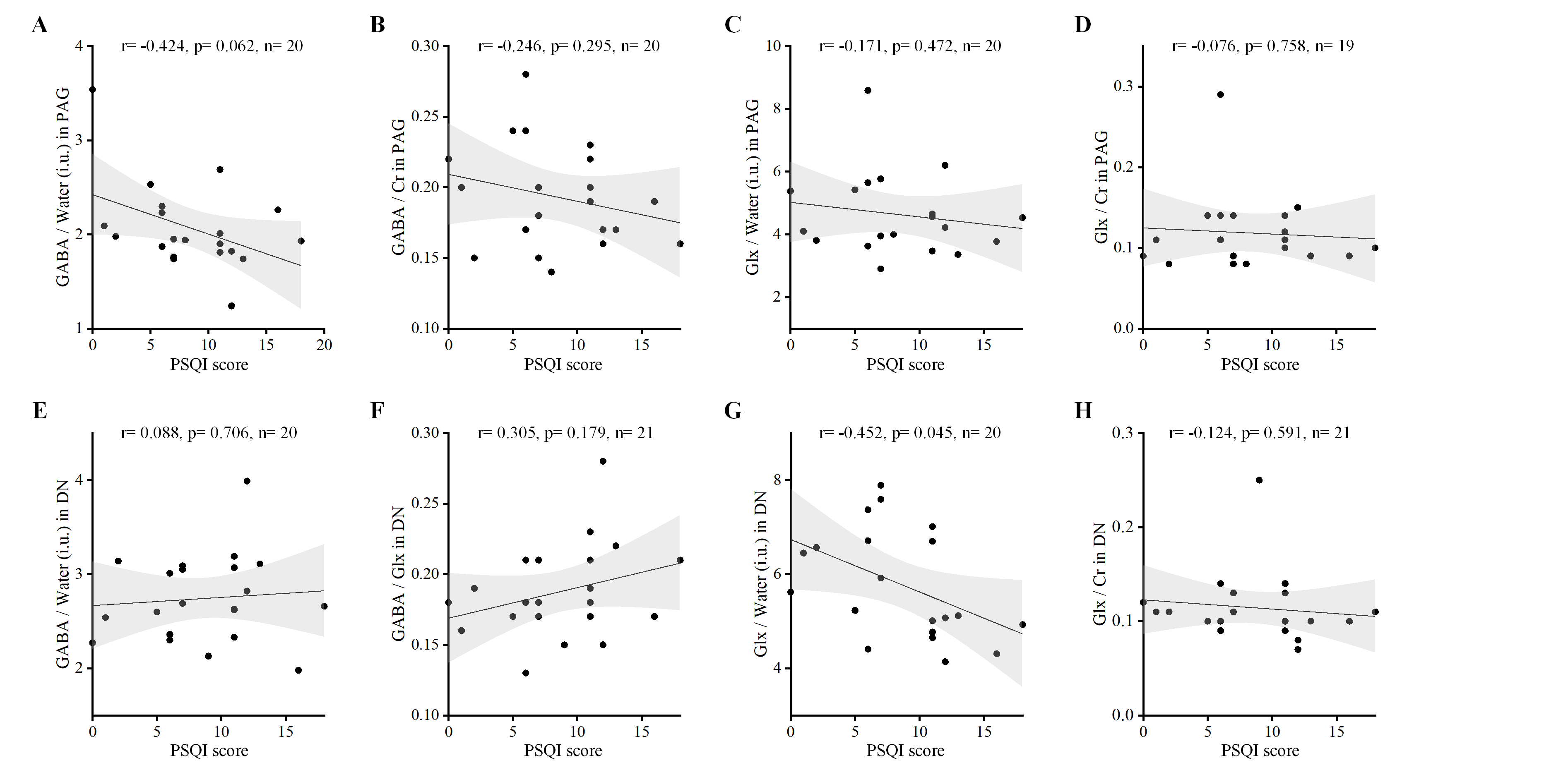

Supplement: Supplementary file 1 — Additional file 1: Fig. S1. Associations between headache frequency and neurochemical levels. (A) and (B) show the correlation between headache frequency and the GABA level in the PAG. (C) and (D) show the correlation between headache frequency and the Glx level in the PAG. (E) and (F) show the correlation between headache frequency and the GABA level in the DN. (G) and (H) show the correlation between headache frequency and the Glx level in the DN. Dots represent participants with migraine. The black regression line represents Pearson’s correlation coefficient (r). Gray shading represents the 95% confidence intervals of the partial correlations. PAG, periaqueductal gray; DN, dentate nucleus; GABA, gamma-aminobutyric acid; Glx, glutamate/glutamine. Fig. S2. Associations between disease duration and neurochemical levels. (A) and (B) show the correlation between disease duration and the GABA level in the PAG. (C) and (D) show the correlation between disease duration and the Glx level in the PAG. (E) and (F) show the correlation between disease duration and the GABA level in the DN. (G) and (H) show the correlation between disease duration and the Glx level in the DN. Dots represent participants with migraine. The black regression line represents Pearson’s correlation coefficient (r). Gray shading represents the 95% confidence intervals of the partial correlations. PAG, periaqueductal gray; DN, dentate nucleus; GABA, gamma-aminobutyric acid; Glx, glutamate/glutamine. Fig. S3. Associations between the MIDAS score and neurochemical levels. (A) and (B) show the correlation between the MIDAS score and GABA level in the PAG. (C) and (D) show the correlation between the MIDAS score and Glx level in the PAG. (E) and (F) show the correlation between the MIDAS score and GABA level in the DN. (G) and (H) show the correlation between the MIDAS score and Glx level in the DN. Dots represent participants with migraine. The black regression line represents Pearson’s correlation coeff [file 10194_2022_1452_MOESM1_ESM.zip › FigureS7.tif]

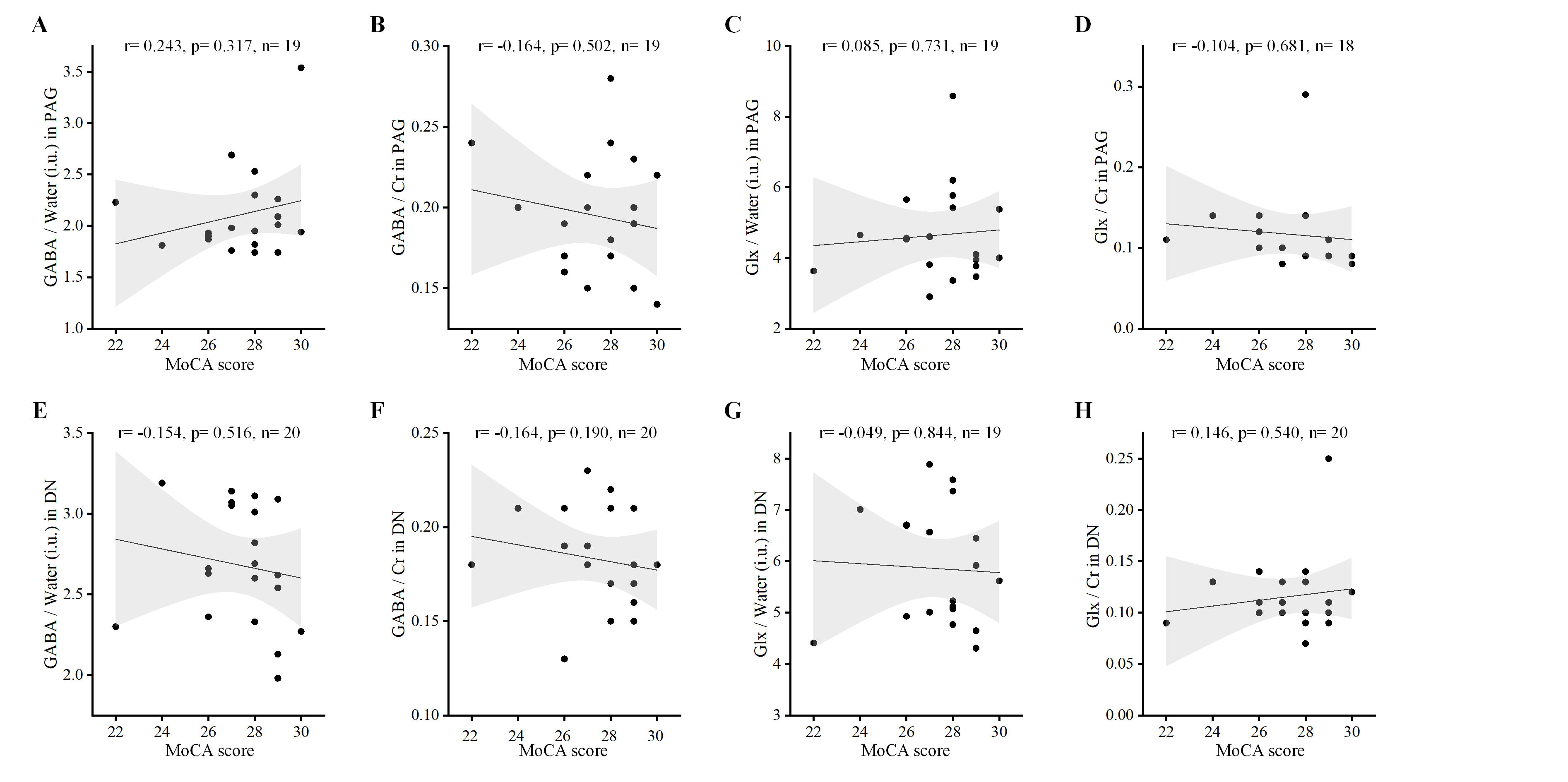

Supplement: Supplementary file 1 — Additional file 1: Fig. S1. Associations between headache frequency and neurochemical levels. (A) and (B) show the correlation between headache frequency and the GABA level in the PAG. (C) and (D) show the correlation between headache frequency and the Glx level in the PAG. (E) and (F) show the correlation between headache frequency and the GABA level in the DN. (G) and (H) show the correlation between headache frequency and the Glx level in the DN. Dots represent participants with migraine. The black regression line represents Pearson’s correlation coefficient (r). Gray shading represents the 95% confidence intervals of the partial correlations. PAG, periaqueductal gray; DN, dentate nucleus; GABA, gamma-aminobutyric acid; Glx, glutamate/glutamine. Fig. S2. Associations between disease duration and neurochemical levels. (A) and (B) show the correlation between disease duration and the GABA level in the PAG. (C) and (D) show the correlation between disease duration and the Glx level in the PAG. (E) and (F) show the correlation between disease duration and the GABA level in the DN. (G) and (H) show the correlation between disease duration and the Glx level in the DN. Dots represent participants with migraine. The black regression line represents Pearson’s correlation coefficient (r). Gray shading represents the 95% confidence intervals of the partial correlations. PAG, periaqueductal gray; DN, dentate nucleus; GABA, gamma-aminobutyric acid; Glx, glutamate/glutamine. Fig. S3. Associations between the MIDAS score and neurochemical levels. (A) and (B) show the correlation between the MIDAS score and GABA level in the PAG. (C) and (D) show the correlation between the MIDAS score and Glx level in the PAG. (E) and (F) show the correlation between the MIDAS score and GABA level in the DN. (G) and (H) show the correlation between the MIDAS score and Glx level in the DN. Dots represent participants with migraine. The black regression line represents Pearson’s correlation coeff [file 10194_2022_1452_MOESM1_ESM.zip › FigureS8.tif]
